# Primary Abdominal Wall Angiosarcoma: A Case Report of Diagnostic Challenges, Local Recurrence, and Metastatic Progression

**DOI:** 10.7759/cureus.110970

**Published:** 2026-06-16

**Authors:** Jeevan Rivera-Díaz, Sara Ocasio-Garí, Isabel Matos-Llovet

**Affiliations:** 1 School of Medicine, Universidad Central del Caribe, Bayamón, PRI; 2 Research and Development, VA Caribbean Healthcare System, San Juan, PRI; 3 Pathology, VA Caribbean Healthcare System, San Juan, PRI

**Keywords:** abdominal wall, angiosarcoma, cd30, cd31, cd34, factor viii, soft tissue sarcoma, vascular neoplasm

## Abstract

Angiosarcoma is an uncommon and aggressive vascular malignancy that may present with nonspecific clinical features, often mimicking benign conditions and leading to delayed diagnosis.

We present a case of a 74-year-old man who presented with a progressively enlarging lesion of the anterior abdominal wall, initially presumed to be a hematoma. Imaging revealed subcutaneous lesions, and surgical excision was performed. Gross examination demonstrated a fungating gray-tan mass with surrounding areas of hemorrhagic discoloration. Histopathologic evaluation revealed a high-grade, poorly differentiated angiosarcoma involving the dermis and subcutaneous tissue, characterized by markedly atypical endothelial cells forming irregular vascular channels, as well as solid and spindle cell areas with numerous mitotic figures. Following initial resection, the patient developed local recurrence requiring multiple subsequent surgical excisions. Despite multimodal management, including repeated surgeries and radiotherapy, the disease progressed, with the development of metastatic lesions involving musculature and bone. Systemic chemotherapy was initiated upon progression.

This case highlights the aggressive clinical course of angiosarcoma, its propensity for local recurrence and distant metastasis, and the diagnostic challenges posed by its variable presentation. Early recognition, histopathologic confirmation, and a multidisciplinary approach are essential for optimal management.

## Introduction

Angiosarcoma is an uncommon and highly aggressive malignancy of vascular endothelial origin, accounting for approximately 2% of all soft tissue sarcomas [1,2]. The incidence of angiosarcoma in the United States (US) has been increasing over the past two decades, with current rates of approximately 3 cases per 1,000,000 person-years [3]. While angiosarcoma can arise in any anatomic location, most cases (72%) occur in cutaneous, subcutaneous, or breast sites, with the head and neck region (particularly the scalp) being the most common location for cutaneous disease [2,3]. Visceral angiosarcomas account for 24% of cases, most commonly involving the liver, heart, and bone, while the remaining percent are located in unknown primary sites [3].

Most cases arise spontaneously as primary angiosarcomas without a clearly identifiable etiology [4]. In contrast, secondary angiosarcoma develops in association with recognized predisposing factors, including ultraviolet light exposure, chronic lymphedema (Stewart-Treves syndrome), and exposure to chemical carcinogens or exogenous toxins such as vinyl chloride, thorium dioxide (thorotrast), arsenic, and anabolic steroids [2,4]. Radiation-associated angiosarcoma has become increasingly recognized, particularly among breast cancer survivors treated with adjuvant radiotherapy, with peak incidence occurring within 10 years after treatment [2,3]. Additional reported associations include hereditary syndromes such as neurofibromatosis, Maffucci syndrome, and Klippel-Trenaunay syndrome, as well as BRCA1/2 mutations [2]. Angiosarcoma has also been linked to foreign bodies, including orthopedic metallic implants and nonfunctioning arteriovenous fistulas in renal transplant recipients [5].

Angiosarcoma of the abdominal wall represents an exceptionally uncommon anatomic location for this malignancy. A large US study in 2024 analyzing 19,289 angiosarcoma cases found that abdominal tumors (including both abdominal wall and intra-abdominal) represented approximately 6% of cases in men and 4% in women, for a total of 298 abdominal cases [3]. The literature on abdominal wall angiosarcoma consists almost entirely of isolated case reports, many of which describe tumors arising in the setting of prior radiation therapy, chronic lymphedema in morbidly obese patients, or following abdominal surgery [6]. Angiosarcoma can present deceptively as a benign-appearing hematoma, ecchymosis, or vascular lesion, often resulting in delayed diagnosis [2,7,8].

We present a case of a 74-year-old man with primary angiosarcoma of the abdominal wall with recurrence and metastasis, highlighting its aggressive nature despite multimodal treatment.

## Case presentation

A 74-year-old man with no past medical history notable for prior malignancies and a body mass index (BMI) of 34.9 presented to the Emergency Department in June 2023 due to a lower abdominal hematoma. The patient denied pruritus, fever, or other constitutional symptoms. Physical examination revealed an ulcerated mass (6-7 cm) with mild blood oozing and tenderness in the localized area for the past three weeks. The patient reported that it had increased slightly in size after a teledermatology consult in April 2023 due to a one-week history of subcutaneous hematoma on the right side of the lower abdomen. The lesion observed during this previous encounter had been attributed to a prior procedure in March 2023, during which the patient underwent bladder chemodenervation with Botulinum A (100 units) for urinary incontinence refractory to oral medication. An abdominal computed tomography (CT) scan was performed, which revealed a focal area of skin thickening involving a pannus in the right lower quadrant with minimal fat stranding and two elevated, peripherally enhancing subcutaneous lesions. The patient underwent surgery in September 2023 for tumor resection.

Gross examination revealed an irregular, fungating gray-tan mass (9.0 × 7.0 × 1.5 cm) arising from the anterior abdominal wall. The lesion was surrounded by an ill-defined, patchy area of purple-red discoloration. The tumor demonstrated superficial erosion and areas of necrosis, consistent with an aggressive vascular neoplasm (Figures 1A, 1B).

**Figure 1:**
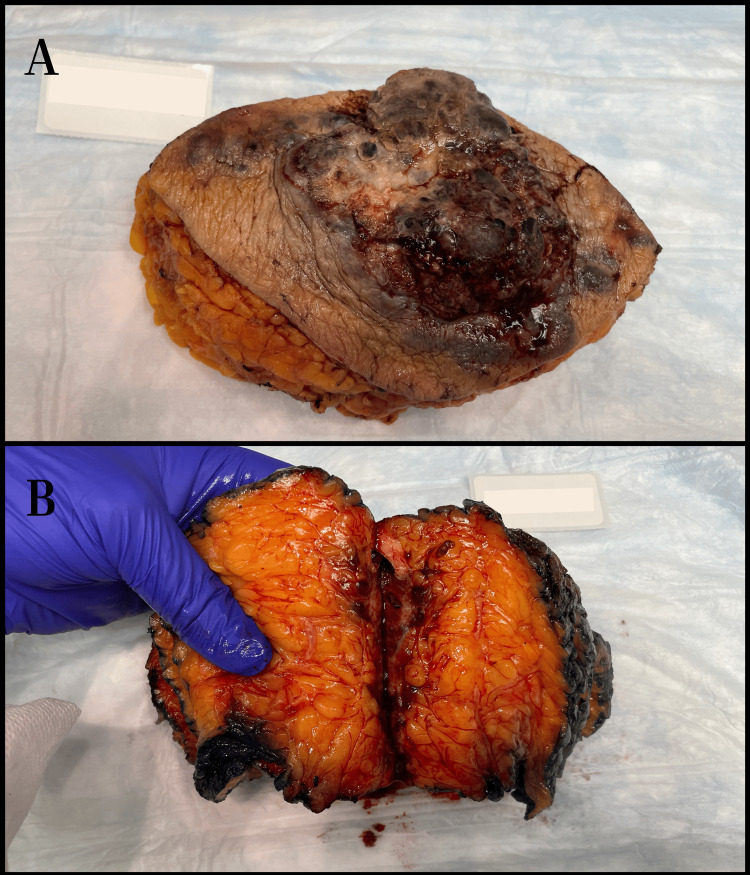
Gross pathology of cutaneous angiosarcoma of the anterior abdominal wall (A) Skin excision demonstrates an irregular, fungating gray-tan mass. Surrounding the lesion is an ill-defined, patchy area of purple-red discoloration. (B) Sectioning of the specimen reveals a poorly circumscribed lesion with areas of hemorrhage and infiltration into the underlying subcutaneous tissue.

Histopathologic evaluation demonstrated a high-grade, poorly differentiated angiosarcoma involving the dermis and subcutaneous tissue. The tumor exhibited a solid sheet-like growth pattern composed of epithelioid, round, polygonal, and spindle cells, admixed with atypical, multilayered endothelial cells forming irregular vessels and cleft-like spaces. Tumor cells displayed marked cytologic atypia with large vesicular nuclei, prominent nucleoli, abundant eosinophilic to amphophilic cytoplasm, and numerous atypical mitotic figures. Additional features included pools of extravasated erythrocytes and hemorrhage dissecting through collagen bundles, multifocal necrosis with cellular swelling and debris, perineural infiltration, and scattered multinucleated giant cells (Figures 2A, 2B, 3A, 3B, 4A, 4B).

**Figure 2:**
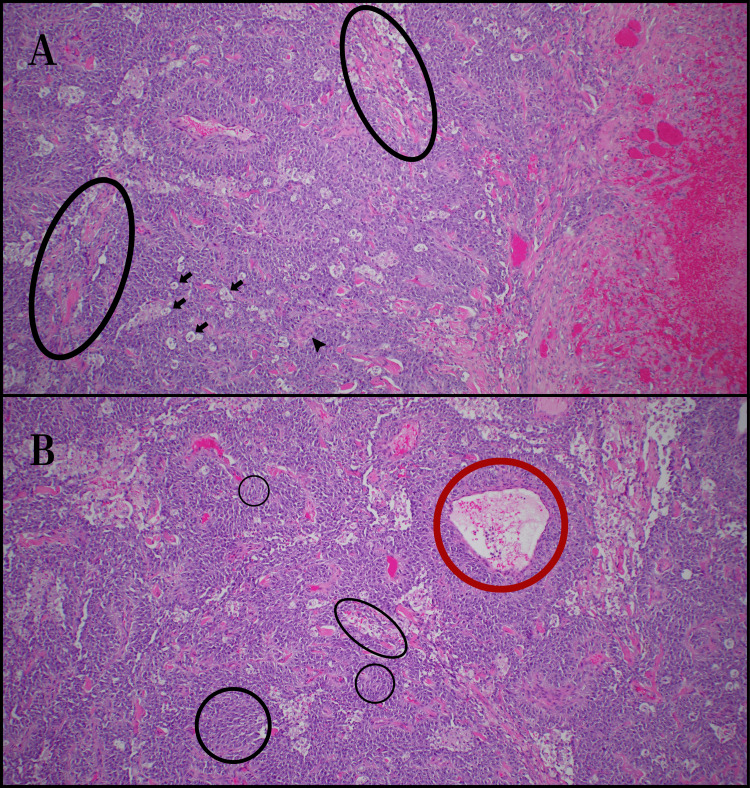
Histopathologic features of abdominal wall angiosarcoma (A) Low-power hematoxylin and eosin (H&E) photomicrograph demonstrating an infiltrative, poorly circumscribed vascular neoplasm composed of irregular anastomosing vascular channels (black oval), a large hemorrhagic area interposed within collagen bundles (right side), tingible-body macrophages (arrows), and giant cells (arrowhead). (B) Low-power magnification shows irregular blood vessels with walls composed of atypical, multilayered, hyperchromatic cells, and cellular infiltration into the lumen (red circle), spindle cell areas (black circle), and extravasated red blood cells (black oval).

**Figure 3:**
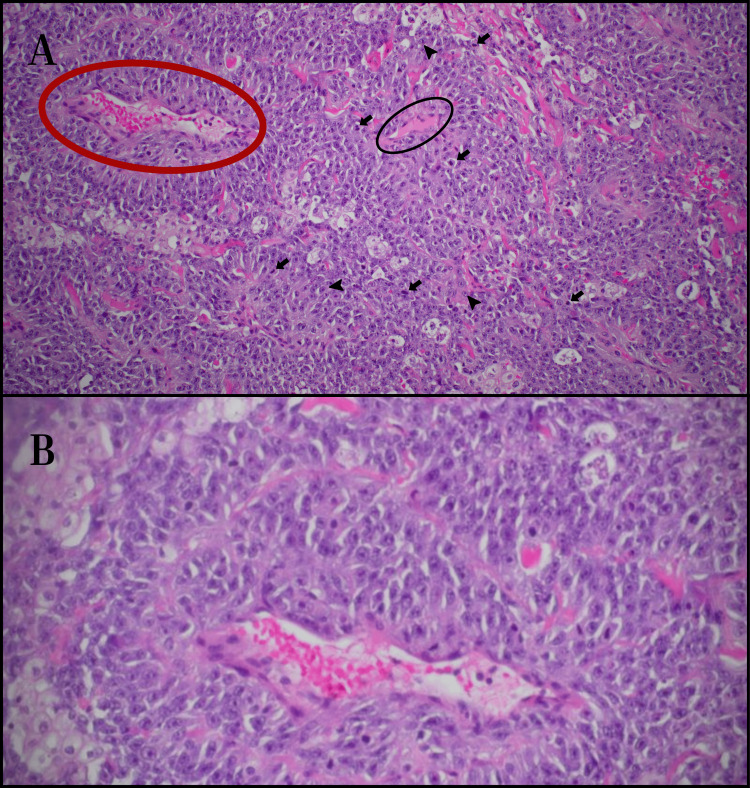
High-power view of abdominal wall angiosarcoma histopathology (A) Intermediate magnification shows atypical endothelial cells with hyperchromatic nuclei lining irregular vascular spaces (red oval), collagen wrapped by endothelial cells (black oval), mitotic figures (arrows), and epithelioid cells (arrowhead). (B) High-power view highlights marked cytologic atypia, pleomorphism, and mitotic activity, with malignant endothelial cells surrounding blood-filled channels, consistent with angiosarcoma.

**Figure 4:**
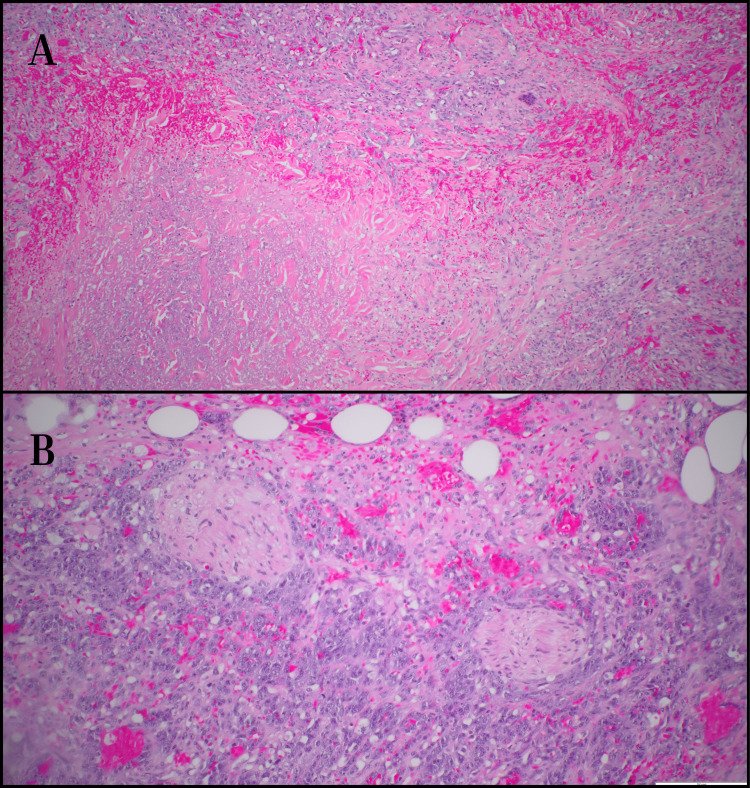
Histopathologic features of tumor necrosis and perineural invasion in angiosarcoma (A) Low-power view demonstrating extensive areas of tumor necrosis with associated hemorrhage and cellular debris within the angiosarcoma. (B) High-power view showing perineural invasion by malignant endothelial cells infiltrating and surrounding peripheral nerve structures, a feature indicative of locally aggressive tumor behavior.

The skin margin of resection was positive for tumor involvement, while the deep margin was free of tumor. Initial staging was classified as pT2N0M0 G3 (stage IIIA) [9]. Immunohistochemical staining demonstrated tumor cell positivity for factor VIII, CD30 in malignant cells, CD31, and CD34 (Figures 5A, 5B, 6A), while epithelial membrane antigen (EMA) was negative (Figure 6B).

**Figure 5:**
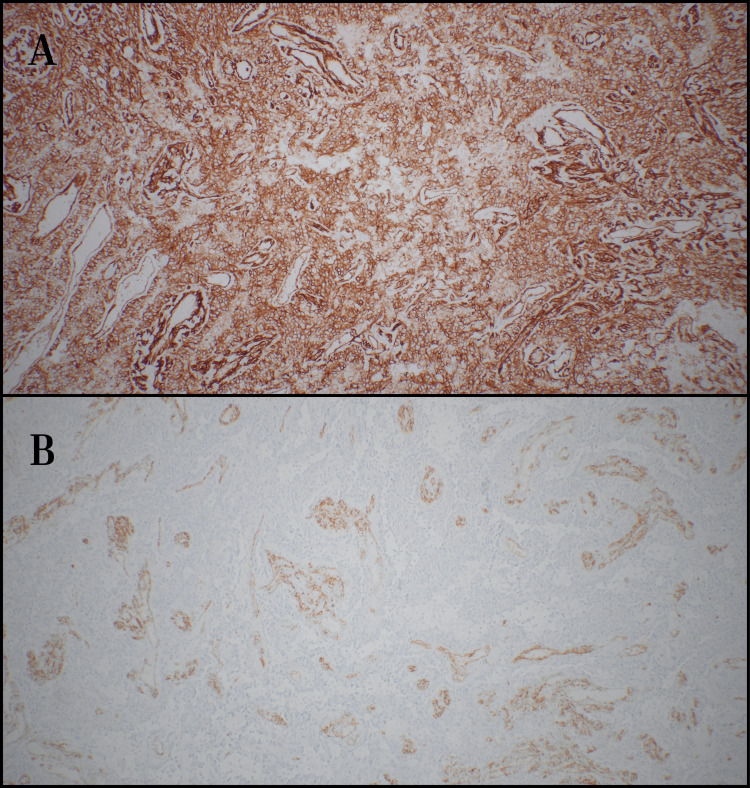
Immunohistochemical profile of abdominal wall angiosarcoma (A) Tumor cells show strong diffuse positivity for CD31, highlighting vascular endothelial differentiation. (B) CD34 demonstrates patchy positivity within neoplastic vascular channels.

**Figure 6:**
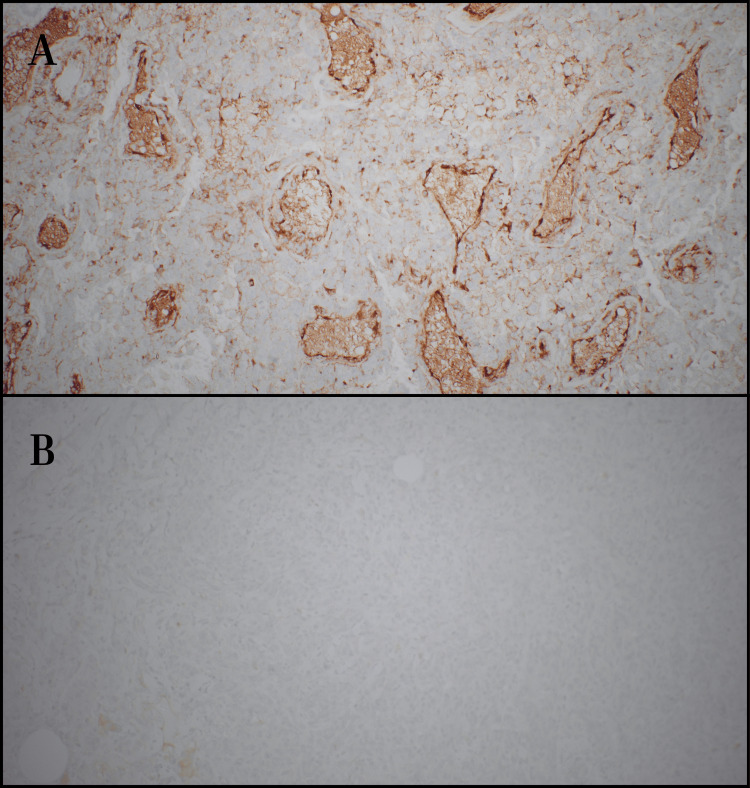
Further immunohistochemical characterization of abdominal wall angiosarcoma (A) Factor VIII-related antigen shows focal positivity, supporting endothelial origin. (B) Epithelial membrane antigen (EMA) is negative, excluding epithelial differentiation and supporting the diagnosis of angiosarcoma.

In November 2023, a positron emission tomography-computed tomography (PET-CT) demonstrated mild postsurgical reactive fat stranding within the subcutaneous compartment of the lower abdomen to the right of the midline, without evidence of fluorodeoxyglucose (FDG)-avid lesions. However, the patient subsequently developed local recurrence of the angiosarcoma, requiring surgical excision in March and July 2024. Histopathologic evaluation of both resections demonstrated findings consistent with the previously described angiosarcoma. Following surgical management, the patient underwent adjuvant radiotherapy, which was completed in November 2024.

Disease progression was subsequently identified on follow-up imaging studies. A CT of the chest, abdomen, and pelvis performed in November 2025 disclosed a persistent subcutaneous soft tissue lesion (1.7 × 1.5 cm), previously 3.4 × 2.8 cm, and a new lobulated enhancing intramuscular lesion within the lateral right flank wall musculature (2.5 × 1.7 cm), consistent with metastatic implantation. A PET-CT performed in January 2026 demonstrated persistent FDG-avid abdominal wall lesions with markedly increased uptake values (SUVmax: 26) (Figure 7A). Additional metastatic progression was identified within the axial skeleton, including multiple hypermetabolic spinal lesions (SUVmax: 20) and a destructive lesion involving the first thoracic vertebra (T1) (Figures 7B, 7C). An additional metastatic lesion was also identified in the right humerus (Figure 7D). Subsequent magnetic resonance imaging (MRI) performed in February 2026 further characterized extensive metastatic involvement, demonstrating abnormal enhancing soft tissue lesions involving the posterolateral paraspinal soft tissues at the level of T1, T2, T5, T7, T10, and the left fourth and sixth posterior ribs (Figure 7E).

**Figure 7:**
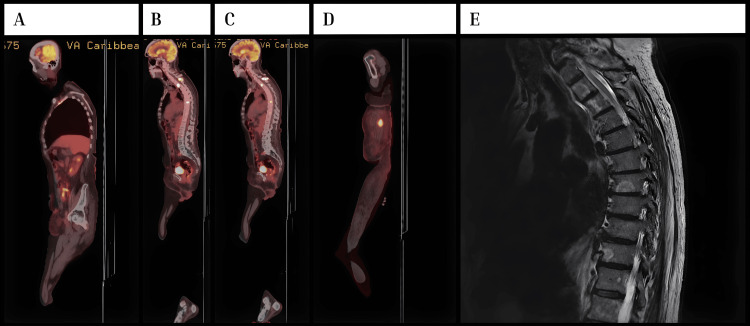
Imaging findings demonstrating metastatic progression of angiosarcoma (A-D) Serial PET-CT images demonstrate progressive FDG-avid lesions involving the abdominal wall, musculature, and skeletal system, consistent with metastatic disease. Increased metabolic activity is noted in the spine and soft tissues. (E) Sagittal MRI of the spine shows osseous involvement with vertebral destruction and associated soft tissue extension, corresponding to metastatic angiosarcoma. PET-CT: positron emission tomography-computed tomography; FDG: fluorodeoxyglucose; MRI: magnetic resonance imaging

Upon disease progression, the patient started chemotherapy with weekly paclitaxel 80 mg/m² on days 1, 8, and 15 of a 28-day cycle. Currently, the patient is alive, continues receiving chemotherapy, and is under consideration for radiotherapy. The patient’s clinical course, including diagnostic evaluation, treatment interventions, and disease progression, is summarized in Figure 8. Given the disease progression, the final staging is T2N0M1 G3 (stage IV) [9].

**Figure 8:**
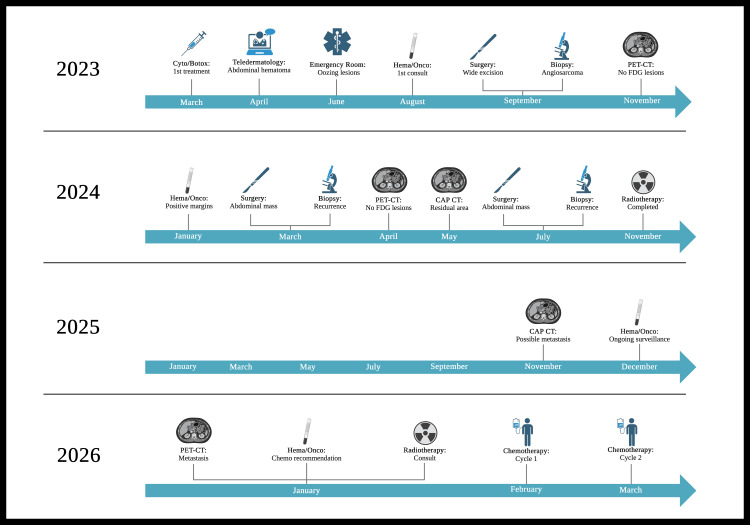
Clinical course and management timeline This timeline outlines the patient’s clinical course from 2023 to 2026, including initial misdiagnosis, subsequent identification of abdominal wall angiosarcoma, and management with surgical excisions, radiotherapy, and surveillance. Despite treatment, the patient developed local recurrences and eventual metastatic disease, leading to initiation of chemotherapy, highlighting the aggressive nature of angiosarcoma and the need for multidisciplinary care. Credit: Jeevan Rivera-Díaz This image is an original author-created schematic using Biorender (BioRender Inc., Toronto, Ontario, Canada) and was not generated using AI (https://BioRender.com/cixq433). PET-CT: positron emission tomography-computed tomography; FDG: fluorodeoxyglucose

## Discussion

Angiosarcoma often presents insidiously and may clinically mimic benign conditions such as hematomas, hemangiomas, ecchymoses, or purplish-red cutaneous lesions, frequently resulting in delayed diagnosis and poor clinical outcomes [7,8]. Diagnosis is further complicated by the rarity of the disease, low clinical suspicion, and overlapping morphologic and immunophenotypic features with other benign and malignant entities. Although endothelial markers are routinely employed, accurate diagnosis generally requires a comprehensive multi-marker panel incorporating both vascular and lymphatic markers (e.g., podoplanin) [4]. Additional diagnostic pitfalls may arise in tumors expressing CD30, particularly epithelioid angiosarcomas, which may morphologically resemble other CD30-positive malignancies such as anaplastic large cell lymphoma, diffuse large B-cell lymphoma, and germ cell tumors [10]. In the present case, the lesion initially appeared clinically as an abdominal hematoma, illustrating the deceptively benign appearance commonly associated with angiosarcoma.

Immunohistochemical evaluation of angiosarcoma commonly relies on endothelial markers such as CD31, CD34, factor VIII-related antigen, erythroblast transformation specific-related gene (ERG), and friend leukemia integration 1 (FLI-1) to confirm vascular differentiation [2,4,8,11]. CD31 and ERG are among the most sensitive markers, typically demonstrating strong membranous and diffuse nuclear staining, respectively [12]. CD34 and factor VIII-related antigen may show more variable expression, with factor VIII-related antigen often displaying weaker and focal staining in poorly differentiated tumors [4,13,14]. FLI-1 is another highly sensitive nuclear endothelial marker that supports vascular origin, although it lacks complete specificity because it may also be expressed in benign vascular lesions and certain non-endothelial neoplasms, including Ewing sarcoma, erythroleukemia, and lymphoma [4,15]. Because these markers are not entirely specific individually, they are interpreted collectively with histopathologic findings to improve diagnostic accuracy. In contrast, EMA, which is commonly expressed in carcinomas, is typically negative in angiosarcoma and assists in excluding epithelial malignancies from the differential diagnosis [16]. CD30 expression may be present in a subset of angiosarcomas, particularly the epithelioid subtype, and can complicate differentiation from other CD30-positive malignancies such as anaplastic large cell lymphoma, diffuse large B-cell lymphoma, and germ cell tumors [10,17]. However, the absence of lymphoid and germ cell immunomarkers, together with the presence of endothelial markers, helps distinguish angiosarcoma from these entities.

The diagnosis of angiosarcoma was favored based on the combination of histopathologic findings and immunohistochemical profile. Atypical fibroxanthoma may mimic angiosarcoma because of its spindle-histiocytoid morphology, prominent vascularization, and hemosiderin deposition; however, the typical absence of ERG, CD31, and CD34 expression helps distinguish it from angiosarcoma [10,11,17]. Likewise, undifferentiated pleomorphic sarcoma may resemble poorly differentiated angiosarcoma because of its patternless proliferation of highly atypical spindle and pleomorphic cells, including bizarre multinucleated giant cells [10,12,18]. However, the presence of vasoformative architecture and endothelial marker expression favors angiosarcoma, whereas the absence of a definitive line of differentiation and only variable CD34 expression supports undifferentiated pleomorphic sarcoma as a diagnosis of exclusion [10,19]. Kaposi sarcoma was considered in the differential diagnosis because of its overlapping vascular features with angiosarcoma, including spindle cell proliferation and expression of endothelial markers such as CD31, CD34, and ERG in certain cases [10,20]. However, Kaposi sarcoma typically occurs in the setting of HHV-8 infection and HIV-associated immunosuppression and characteristically demonstrates vascular clefts, hemosiderin deposition, intracytoplasmic hyaline globules, and only minimal cytologic atypia [12]. The presence of marked pleomorphism, multilayered solid growth, epithelioid morphology, and atypical mitotic activity supports angiosarcoma over Kaposi sarcoma. Moreover, anastomosing hemangioma can be excluded because of the pronounced cytologic atypia, infiltrative growth, and aggressive histologic features absent in benign vascular lesions [10,21,22]. Poorly differentiated carcinoma and other CD30-positive malignancies may also be considered; however, the strong expression of endothelial markers, together with the absence of epithelial marker expression, supported angiosarcoma over epithelial or lymphoid neoplasms [10].

Genomic studies of angiosarcoma have identified TP53 mutations and MYC amplification as the most frequent molecular alterations, occurring in approximately 20%-30% of cases [23,24]. TP53 mutations are particularly common in head and neck angiosarcomas, whereas MYC amplification is strongly associated with secondary angiosarcomas, especially those related to radiation exposure and chronic lymphedema [4,24]. Although MYC amplification is considered a sensitive and highly specific marker for secondary angiosarcoma, it has also been reported in a small subset of primary angiosarcoma [4,25]. Nonetheless, alterations involving angiogenic pathways, including KDR and PIK3CA mutations, are more commonly observed in primary angiosarcomas [23].

Chronic lymphedema is a well-established risk factor for angiosarcoma, a phenomenon known as Stewart-Treves syndrome [26]. Secondary angiosarcoma has been particularly associated with chronic lymphedema following breast cancer treatment, often in conjunction with prior radiation therapy [27]. Obesity is strongly linked to the development and severity of lymphedema, with risk increasing substantially at higher BMI levels (>40) due to obesity-induced lymphatic dysfunction [28,29]. Clinically, lymphedema progresses from a sensation of heaviness and pitting edema to fibrotic tissue changes and severe cutaneous thickening in advanced stages [30]. Diagnosis is primarily clinical, based on history and physical examination findings [31]. In the present case, however, the patient had class I obesity without clinical evidence of lymphedema, making Stewart-Treves syndrome less likely as a contributing factor. 

Treatment of localized angiosarcoma typically involves multimodal therapy, with surgical resection with wide margins serving as the cornerstone of treatment [32]. Yet, achieving negative margins is often difficult due to the infiltrative and often multifocal nature of these tumors; therefore, adjuvant radiotherapy is commonly employed to improve local disease control [2]. In advanced or metastatic disease, systemic therapy becomes the primary treatment modality, with anthracycline-based regimens (particularly doxorubicin) and taxanes (paclitaxel or docetaxel) representing preferred first-line options [32-34]. Increasing evidence supports the efficacy of weekly paclitaxel, especially in cutaneous and radiation-associated angiosarcoma, with comparative studies demonstrating higher objective response rates relative to doxorubicin, while outcomes in non-cutaneous angiosarcoma appear similar between both agents [35-37]. Angiosarcoma is notably one of the few soft tissue sarcomas responsive to single-agent taxane therapy [38]. Likewise, weekly paclitaxel offers a more favorable tolerability profile for many patients because it lacks the cumulative cardiotoxicity associated with doxorubicin and is generally associated with lower hematologic toxicity, although higher-dose paclitaxel regimens (135-175 mg/m²) may still produce significant neutropenia and peripheral neuropathy [30,39-41]. Emerging therapeutic options include anti-angiogenic tyrosine kinase inhibitors, such as pazopanib, and immune checkpoint inhibitors, particularly in cutaneous angiosarcoma [32-34]. In the present case, the patient underwent adjuvant radiotherapy following his third surgical resection and was later initiated on paclitaxel after the development of muscle and osseous metastases. An important consideration in this case is whether earlier initiation of systemic therapy or the incorporation of concurrent chemoradiation during repeated local recurrences might have altered the clinical course. Although this cannot be determined retrospectively, earlier systemic treatment may merit consideration in selected patients with recurrent, high-grade, or multifocal disease because of the known risk of occult metastatic progression.

The prognosis of angiosarcoma remains poor despite advances in treatment, with reported five-year survival rates ranging from 10% to 35% and median survival often less than two years, particularly in visceral disease [2,42-45]. Prognostic factors associated with worse outcomes include advanced age (≥65 years), tumor size (≥5 cm), high histologic grade (G3), presence of distant metastases at diagnosis, osseous metastatic progression, and certain anatomic locations, particularly visceral and head-and-neck involvement [2,42,46-49]. MYC amplification, particularly in secondary angiosarcoma, as well as increased expression of p53 and c-MYC, have also been linked to aggressive tumor behavior, increased metastatic potential, and reduced survival [4]. Initial staging in this case was classified as pT2N0M0 G3 (stage IIIA), later progressing to T2N0M1 G3 (stage IV). However, conventional pathologic staging may have limited applicability in angiosarcoma because of its distinct biologic behavior compared with other soft tissue sarcomas [50]. Although our patient experienced an aggressive clinical course with recurrent and metastatic disease, his survival beyond two years highlights the heterogeneity of outcomes in angiosarcoma and may reflect factors such as tumor biology, repeated local treatment, and multidisciplinary oncologic management.

Several clinical features should raise suspicion for malignancy and warrant tissue sampling. Concerning findings include the absence of a clear history of trauma, a presumed hematoma that persists or enlarges beyond four weeks, and soft tissue masses that are ≥5 cm, deep to the fascia, rapidly growing, firm, or fixed to adjacent structures [51]. Lesions without a definitive diagnosis should undergo close surveillance or biopsy. Percutaneous core needle biopsy is the preferred initial diagnostic approach, ideally performed with image guidance to target viable enhancing tissue and avoid necrotic areas, whereas open incisional biopsy may be considered if core needle biopsy is nondiagnostic [32]. 

## Conclusions

Angiosarcoma is an aggressive vascular malignancy that frequently presents with nonspecific features mimicking benign conditions, leading to diagnostic delays. Even in the absence of known risk factors for angiosarcoma, early biopsy of non-resolving vascular-appearing lesions is critical for timely diagnosis. Histopathological examination with immunohistochemical confirmation using endothelial markers such as ERG, CD31, CD34, and factor VIII-related antigen is essential for differentiating angiosarcoma from other tumors.

This case demonstrates the typical aggressive behavior of angiosarcoma, with early local recurrence and metastatic progression despite multimodal therapy including surgical resection and adjuvant radiotherapy. Due to its variable presentation, early recognition through heightened clinical suspicion, prompt histopathologic confirmation, and coordinated multidisciplinary management are essential for optimizing patient outcomes. Given its associated poor overall prognosis, continued research focusing on quicker diagnostic strategies and targeted therapies is critical to improving patient outcomes.
